# Nonenzymatic Glucose Sensor Based on In Situ Reduction of Ni/NiO-Graphene Nanocomposite

**DOI:** 10.3390/s16111791

**Published:** 2016-10-26

**Authors:** Xiaohui Zhang, Zheng Zhang, Qingliang Liao, Shuo Liu, Zhuo Kang, Yue Zhang

**Affiliations:** 1 State Key Laboratory for Advanced Metals and Materials, School of Materials Science and Engineering, University of Science and Technology Beijing, Beijing 100083, China; Zhangxiaohui@crrcgc.cc (X.Z.); liao@ustb.edu.cn (Q.L.); ruodehaner123@126.com (S.L.); zhuokang@ustb.edu.cn (Z.K.); 2 CRRC Institute, Beijing 100070, China; 3 Beijing Municipal Key Laboratory for Advanced Energy Materials and Technologies, Beijing 100083, China

**Keywords:** nonenzymatic, glucose, graphene, biosensor, in situ

## Abstract

Ni/NiO nanoflower modified reduced graphene oxide (rGO) nanocomposite (Ni/NiO-rGO) was introduced to screen printed electrode (SPE) for the construction of a nonenzymatic electrochemical glucose biosensor. The Ni/NiO-rGO nanocomposite was synthesized by an in situ reduction process. Graphene oxide (GO) hybrid Nafion sheets first chemical adsorbed Ni ions and assembled on the SPE. Subsequently, GO and Ni ions were reduced by hydrazine hydrate. The electrochemical properties of such a Ni/NiO-rGO modified SPE were carefully investigated. It showed a high activity for electrocatalytic oxidation of glucose in alkaline medium. The proposed nonenzymatic sensor can be utilized for quantification of glucose with a wide linear range from 29.9 μM to 6.44 mM (R = 0.9937) with a low detection limit of 1.8 μM (S/N = 3) and a high sensitivity of 1997 μA/mM∙cm^−2^. It also exhibited good reproducibility as well as high selectivity.

## 1. Introduction

Diabetes is a worldwide public health problem and one of the leading causes of death and disability [1]. The diagnosis and management of diabetes mellitus requires serious monitoring of blood glucose levels. Since Clark and Lyons first proposed the concept of glucose enzyme electrodes in 1962 [2], glucose sensors have become the most important biosensors, not only in research field but also in the market for the past 50 years [3]. Electrochemical sensors based on glucose dehydrogenase and glucose oxidase have been extensively studied due to their high selectivity and good sensitivity towards glucose [4,5]. The inevitable disadvantages of enzymes still limit the measurement and analytical application of enzyme electrodes based glucose sensors, such as the chemical or thermal instabilities originated from the intrinsic nature [6,7,8].

Nonenzymatic electrocatalysis to direct oxidation of glucose is a feasible alternative technology that is free from the mentioned drawbacks. It depends on the current response of glucose oxidation directly at the electrode surface to achieve high sensitivity and the selectivity. Generally, the key electrode materials of nonenzymatic sensors consist of the nanostructures of transition metals and their alloys, such as copper [9], platinum [10], palladium [11], nickel [12], etc. Compared with these metals structures, Ni and its chemical compound, such as Ni nanowire array [13], NiO hollow microsphere [14], and Ni(OH)_2_ nanoplates [15], are proven to allow production of high sensitivity glucose sensors at low cost [16,17]. Further, the oxidation processes of glucose, which is catalyzed by nickel, are through the formation of a high-valent, oxyhydroxide species (NiOOH) in alkaline medium [13].

Graphene, a one atom thick material consisting of sp2 bonded carbon with a honeycomb structure, has drawn a vast amount attention since its discovery [18] ascribing to its excellent physical and chemical properties [19]. Graphene’s high surface area, easy functionalization, excellent electron transfer, and good biocompatibility, make it become a super platform for biological analysis and detection [20,21,22]. The application of graphene in developments of nonenzymatic sensors would provide high reliability and stability for glucose analysis and monitoring. Thus, recently, researchers started to focus on Ni-graphene hybrid for enzyme free glucose detection [23,24]. CuNiO nanocubic modified graphene sheets showed wide linear range up to 16 mM [25]; Ni(OH)_2_ nanoplates on reduced graphene oxide (rGO) exhibited low detection limit of 0.6 μA.

With the motivation of developing a miniaturized lab-on-chip device for routine glucose measurement, we employed replaceable and disposable screen printed electrodes (SPEs) to construct enzyme-free glucose sensors, modified by Ni/NiO-rGO nanocomposite. The synthesis of the nanocomposite is initiated by electrostatic process and formation of self-assembled nanocomposite precursors of negatively charged graphene oxide (GO) and positively nickel cations (Ni^2+^). The resulting nanocomposite facilitated electron transfers and endowed high electrocatalytic activity for non-enzymatic glucose sensing. It provided high sensitivity, rapidity, simplicity and reproducibility advantages.

## 2. Experimental Methods

### 2.1. Regents and Apparatus

Graphene oxide (GO) dispersion was commercial obtained from Nanjing XFNANO Materials Tech Co., Ltd. (Nanjing, China); Glucose and nickel acetate (Ni(CH_3_COOH)_2_) were purchased from Sinopharm Chemical Reagent Co., Ltd. (Shanghai, China); 5% Nafion ethanol solution, lactic acid (LA), uric acid (UA), bull serum albumin (BSA), and ascorbic acid were obtained from Sigma-Aldrich; and Screen printed electrodes (SPEs, AC1.W4.RS) were constructed by BVT Technologies.

The morphologies and structures of rGO sheets were characterized by employing FESEM (Zeiss, SUPRA-55), Raman spectrometer (Jobin-Yvon, JY-HR800, 514 nm), and XPS (Axis UltraDLD). The cyclic voltammetric and amperometric response measurements of Ni/NiO-Nafion-rGO/SPEs were investigated by advanced electrochemical interface (Solartron Analytical, SI 1287).

### 2.2. Rinse and Activation of SPEs

Before reducing process, SPEs were rinsed under ultrasonic agitation for 3 min by the mixture of sulfuric acid (volume fraction 5%), hydrochloric acid (volume fraction 5%), potassium hydroxide (0.5 mol/L), and phosphate buffer solution (PBS, 0.1 mol/L, pH = 7.4). To accelerate the electron transfer and enhance the electrochemical activity of SPEs, electrochemical activation [26] was conducted under 1.5 V for 180 s. 

### 2.3. In Situ Synthesis of Ni/NiO-rGO Modified SPEs

**Scheme 1:**
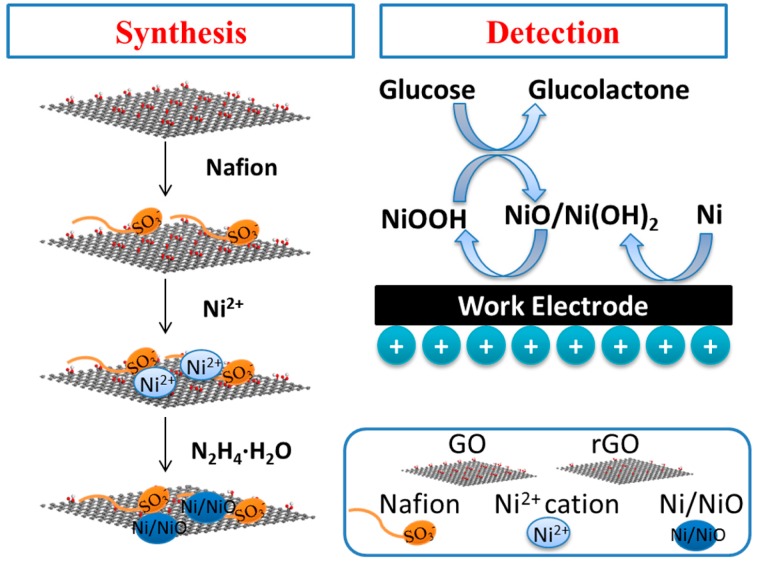
The in situ synthesis of the Ni/NiO-rGO nanocomposite and the detection principle of the electrocatalysis of glucose.

The mixture of Nafion solution (5 wt.%, 100 μL) and GO dispersion (0.5 g/L, 100 μL) was incubated for 30 min at room temperature to format GO-Nafion hybrid. Nafion was utilized to the in situ synthesis of Ni/NiO-rGO nanocomposite for introducing the negative charges for the electrostatic and self-assembled precursors. Then, nickel acetate solution (Ni(CH_3_COOH)_2_, 0.1 mol/L, 60 μL) is added to GO-Nafion hybrid (20 μL) and incubated for 60 min. During such incubation, Ni^2+^ would chemically absorb on GO sheets. Then, the work electrode of SPE was immersed in the proposed Nafion-GO-Ni^2+^ mixture for 30 min to immobilize GO and Ni^2+^. Finally, hydrazine hydrate (N_2_H_4_∙H_2_O, 10 mmol/L) was exerted on the work electrode of SPEs for 20 h at room temperature to in situ reduce GO and Ni^2+^. The formation mechanism of in situ synthesized Ni/NiO-rGO-Nafion nanocomposite is shown in Scheme 1.

## 3. Results and Discussion

### 3.1. Morphology and Structure Characterization

**Figure 1:**
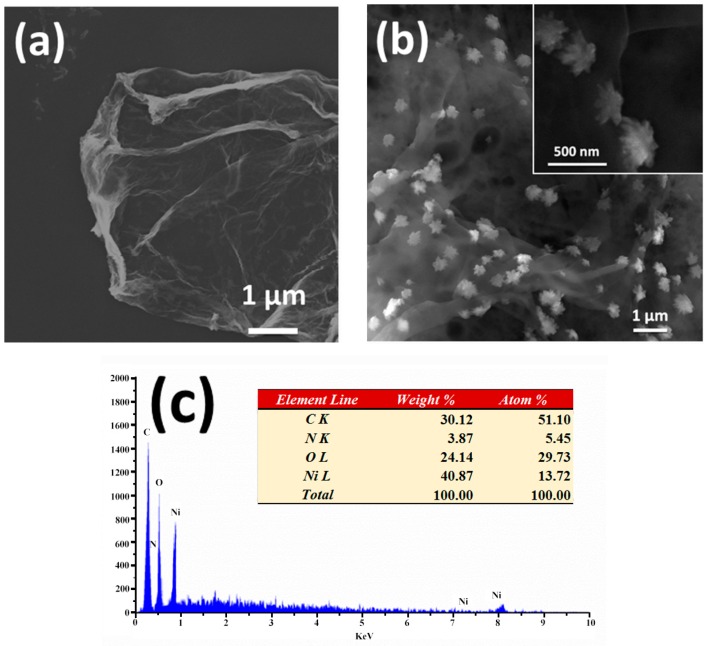
(**a**) FESEM morphologies of GO; and (**b**) Ni/NiO-rGO; and (**c**) surface EDS spectrum of Ni/NiO-rGO.

FESEM morphologies of GO and in situ reduced Ni/NiO-rGO nanocomposite are shown in Figure 1a,b. Both GO and rGO sheets are in micrometer size and obviously wrinkled structures. Flower-like Ni/NiO nanoparticles with the size of 300–400 nm (insert Figure 1b) are dispersed on rGO sheet homogeneously. Elemental composition of Ni/NiO-rGO nanocomposite was determined by surface EDS (Figure 1c). Signature peaks of C, O, and Ni were characterized, and a weak peak indicating N from the reduce agent hydrazine hydrate is also obtained. 

**Figure 2:**
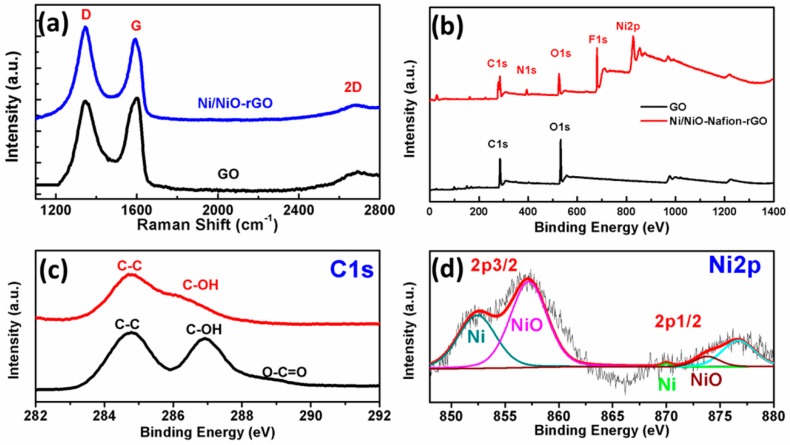
Structure characterization of the in-situ reduction of Ni/NiO-rGO nanocomposite: (**a**) Raman spectra of GO (black curve) and Ni/NiO-rGO nanocomposite (blue curve); (**b**) Entire XPS spectra of GO (black curve) and Ni/NiO-Nafion-rGO nanocomposite (red curve); (**c**) XPS spectra of C1s for GO and Ni/NiO-Nafion-rGO; and (**d**) XPS spectrum of Ni2p for Ni/NiO-Nafion-rGO.

The structural characterization of GO and Ni/NiO-rGO sheets was further performed by analyzing the Raman spectra, as shown in Figure 2a. The well-defined D bands (~1350 cm^−1^), characteristic of the first-order scattering from a zone-boundary phonon [27], are strong in both GO and Ni/NiO-rGO samples, which indicates the defective crystal structures for both GO and Ni/NiO-rGO. Besides, 2 cm^−1^ negative shift observed after modified progress, suggesting the successful reduction of GO [28]. G band (~1600 cm^−1^) is assignable to the E2g phonon of sp2 carbon atoms. The intensity ratio of D to G band (I_D_/I_G_) reflects the defect density of graphene sheets. I_D_/I_G_ increased from 0.97 to 1.10 after reduction, demonstrating the defects formation during reduction and Ni/NiO assembling. 2D bands (~2670 cm^−1^), representative of the second-order zone-boundary phonons, are relatively week in both GO and Ni/NiO-rGO, indicating the multilayers structures of grapheme [29].

The functional groups and the Ni content in graphene sheets were conducted by XPS spectra (Figure 2b–d). As shown in the entire XPS spectra (Figure 2b), peaks of C1s and O1s are relevantly strong in both GO and Ni/NiO-Nafion-rGO. Compared to GO, additional peaks of N1s, F1s and Ni2p resulting from hydrazine hydrate residual, Nafion and Ni element in Ni/NiO-Nafion-rGO are observed. The atomic content of C and O in GO sheets is calculated to be 69.8% and 39.2%, respectively. Accordingly, the atomic content of C, N, O, F and Ni in Ni/NiO-Nafion-rGO are determined to be 52.8%, 3.4%, 10.4%, 7.2% and 26.2%, respectively. This reveals that the atomic ratio of C and O (C:O) increased from 1.76 to 5.08 after reduction, which is also supported by Raman spectra results. Moreover, the oxygen related functional group, C–OH and O–C=O, are obviously reduced in Ni/NiO-Nafion-rGO compared to GO (Figure 2c). The great number of surface oxygen functional groups on GO sheets can play the role of cation adsorption and nucleation sites for metal nanoparticles [30]. Accordingly, the effective deoxygenating of GO by the in situ reduction process improved the electrical conductivity of graphene that can be the supportive material in catalyst layers [9].

Ni2p spectrum of Ni/NiO-Nafion-rGO is shown in Figure 2d. The main satellite peaks at 857.2 and 876.7 eV are generally caused by multi-electron excitation [31]. According to these “shake-up” peaks, four separated peaks, located at 852.4, 857.2, 870.0 and 873.8 eV, represent metallic Ni (2p3/2), NiO (2p3/2), metallic Ni (2p1/2) and NiO (2p1/2), respectively [32,33]. The as-synthesized Ni/NiO-Nafion-rGO nanocomposite would greatly affect the electrochemical properties of modified SPEs in alkaline medium.

### 3.2. Electrochemical Characterizations

**Figure 3:**
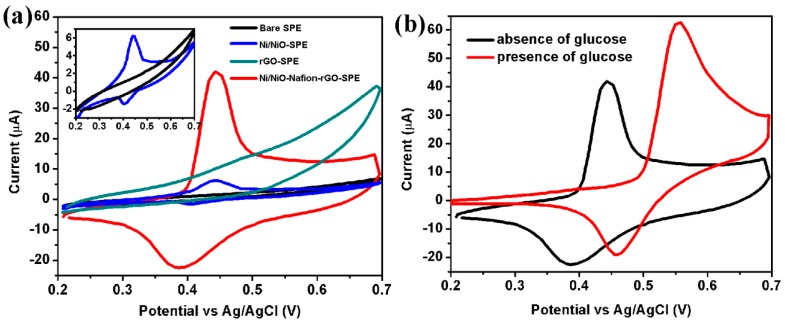
(**a**) Cyclic voltammograms of bare SPE, rGO-SPE, Ni/NiO-SPE and Ni/NiO-rGO-SPE in 0.1 M NaOH at scan rate of 50 mV/s (insert show the magnification of bare SPE and Ni/NiO-SPE); (**b**) Cyclic voltammograms of Ni/NiO-Nafion-rGO modified SPE in absence (black curve) and presence (red curve) of 3 mM glucose in 0.1 M NaOH.

Cyclic voltammograms (CVs) are performed on bare SPE, Ni/NiO-SPE, rGO/SPE, and Ni/NiO-Nafion-rGO-SPE, as shown in Figure 3a. In the presence of 0.1 M NaOH, the current of rGO-SPE is considerably enhanced compared to the bare SPE. Moreover, no redox peak is observed towards both bare SPE and rGO-SPE, suggesting no oxidation-reduction reaction occurred in alkaline medium without nickel catalyzing. A pair of asymmetric redox peak at 0.450 V and 0.402 V is observed for Ni/NiO-SPE (insert of Figure 3a). As Ni/NiO-rGO incorporates onto the SPEs, the current is significantly increased comparing to Ni/NiO-SPE, and a pair of well-defined asymmetric redox peak at 0.444 V and 0.387 V is observed, which could be attributed to the electron transfer between Ni (II) and Ni (III) [14]:

NiO + OH^−^ → NiOOH + e^−^ (1)

Furthermore, the metallic Ni on rGO sheets would experience the reaction of Ni (0) to Ni (II), and then Ni (II) to Ni (III) [34]:

Ni + 2OH^−^ → Ni(OH)_2_ + 2e^−^ (2)

Ni(OH)_2_ + OH^−^ → NiOOH + H_2_O + e^−^ (3)

The rGO sheets contribute to improve the electrochemical activity of SPEs, and Ni/NiO composite is mainly responsible to provide electrocatalysis activity in alkaline environment. 

The CV measurement was further applied to investigate the electrochemical behavior of Ni/NiO-Nafion-rGO/SPE in 0.1 M NaOH absent and present 3 mM glucose (Figure 3b). When glucose is added into the alkaline ambience, the anodic peak current is apparently enhanced accompanied with a positive shift of peak potential from 0.444 V to 0.558 V, while the cathodic peak abated with a positive shift from 0.387 V to 0.459 V, which suggests excellent electrocatalytic activity of Ni/NiO-Nafion/rGO in glucose oxidation process. The oxidation of glucose to glucolactone is electrocatalyzed by the Ni(III)/Ni(II) redox couple according to the following electrochemical reaction [23]:

NiOOH + glucose → Ni(OH)_2_ + glucolactone (4)

The enhancement of redox potential is due to adsorption of glucose and the oxidized intermediates on the active sites of Ni/NiO, which slows down the kinetics of the above reaction. Accordingly, the cathodic peak current reducing is ascribed to the consumption of Ni^3+^ (NiOOH), which is induced by the oxidation of glucose [14].

**Figure 4:**
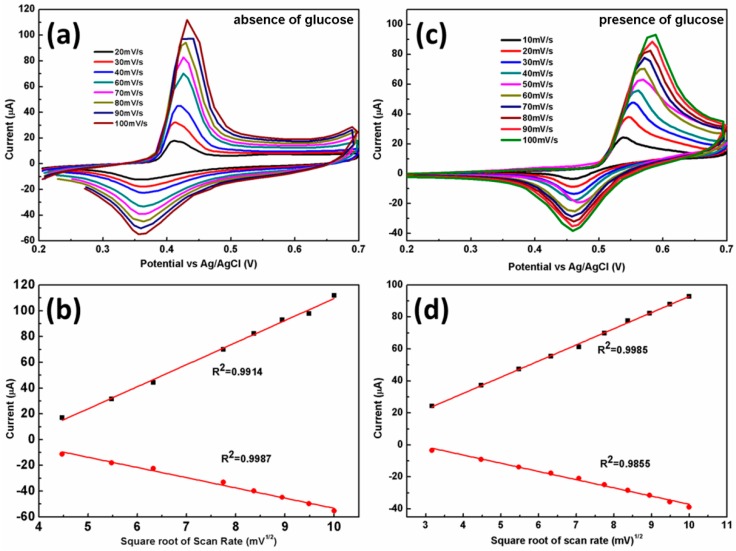
Cyclic voltammograms of Ni/NiO-Nafion-rGO modified SPE in absence (**a**) and presence (**c**) of glucose in 0.1 M NaOH with different scan rate; and plots of peaks current (Ip) vs. square root of scan rate in absence (**b**) and presence (**d**) of glucose in 0.1 M NaOH.

Further investigation of electron transfers behaviors of Ni/NiO-Nafion-rGO/SPE is performed using CV technique under various scan rates in absence and presence of glucose in alkaline condition, as shown in Figure 4. With the absence of glucose (Figure 4a), both cathode and anode current peaks linearly increase with the square root of scan rates and excellent correlation coefficients (0.9914 and 0.9987) achieves (Figure 4b). It reveals that Reaction (1) is a quasi-reversible electron transfer and diffusion controlled process that depends on OH^−^ diffusion to the electrode surface. With the presence of glucose, the anodic peaks show a positive shift along the scan rate from 0.537 V to 0.585 V, while the cathodic peaks maintained at ~0.459 V (Figure 4c). The separation of cathode and anode potential increased with scan rate, which implied that the reaction rate decreased with the presence of glucose. Currents of both cathode and anode peak increase linearly with the square root of scan rate, indicating a diffusion controlled process.

### 3.3. Performance of Ni/NiO-Nafion-rGO/SPEs Nonenzymatic Glucose Sensors

**Figure 5:**
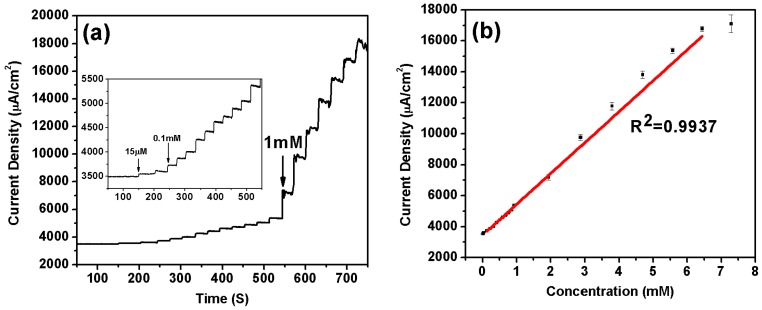
(**a**) Amperometric response of Ni/NiO-Nafion-rGO nanocomposite modified SPE in a series of concentrations of glucose at an applied potential of 0.55 V (insert show the magnification of low glucose concentration); (**b**) Linear calibration curve to the amperometric response.

The amperometric response of constructed nonenzymatic sensor is evaluated with successive addition of glucose at an applied potential of 0.55 V. As shown in Figure 5a, the current response increases with the addition of various concentration of glucose. A rapid response of the nonenzymatic catalyzed glucose less than 2 s (reaching 95% of the steady-state current) is achieved. It can detect glucose linearly from 29.9 μM to 6.44 mM (R = 0.9937) with a detection limit of 1.8 μM (at S/N = 3), as shown in Figure 5b. The sensitivity is calculated to be 1997 μA/mM∙cm^−2^. Compared to the performance of other nonenzymatic glucose sensors based on graphene and transition metals (Table 1), the as-fabricated sensor shows an excellent sensitivity and a suitable detection range for human blood monitoring [35]. Besides, the sensitivity of the nonenzymatic sensors is also better than the reported enzymatic sensors [36].

**Figure 6:**
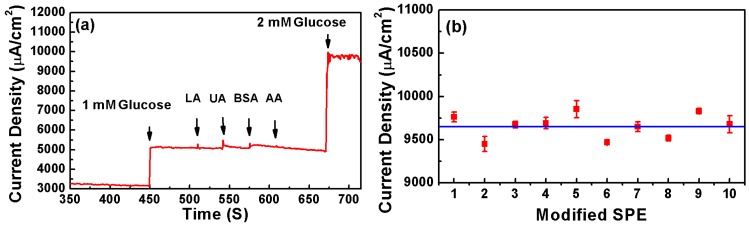
(**a**) Selectivity measurement of amperometric responses to successive addition of 1 mM glucose, 0.1 mM LA, 0.1 mM UA, 0.1 mM BSA, 0.1 mM AA, and 2 mM glucose; (**b**) Reproducibility measurement of 10 amperometric response towards 3 mM glucose.

The anti-interference experiment of Ni/NiO-Nafion-rGO/SPE is investigated by successive addition of 1 mM glucose, 0.1 mM LA, 0.1 mM UA, 0.1 mM BSA, 0.1 mM AA, and 2 mM glucose at an applied potential of 0.55 V, as shown in Figure 6a. The addition of above inferences apparently did not induce current response, while current response to 3 mM glucose is ~9600 μA/cm^2^.

The reproducibility of Ni/NiO-Nafion-rGO-SPE is investigated from the current response to 3 mM glucose at 10 individual devices, as shown in Figure 6b. A good reproducibility with relative standard deviation (RSD) of 1.46% is achieved. Furthermore, the average current response to 3 mM glucose is calculated to be 9624 μA/cm^2^.

## 4. Conclusions

In situ synthesized Ni/NiO-rGO nanocomposite modified SPEs were applied to the sensitively detection of glucose free of enzyme. This Ni/NiO-Nafion-rGO/SPE nonenzymatic glucose sensor shows a high electrochemical activity for electrocatalytic oxidation of glucose in alkaline medium. The constructed nonenzymatic biosensor presented a wide linear range from 29.9 μM to 6.44 mM (R = 0.9937), a low detection limit of 1.8 μM (S/N = 3), a fast response of less than 2 s, and a sensitivity as high as 1997 μA/mM∙cm^−2^. Besides, good reproducibility and high selectivity were also demonstrated. The proposed sensor can be facilely fabricated, which allows for economical and miniaturized construction of lab-on-chip devices. It also reveals a great potential for graphene based materials to be utilized in surface and interface science.

## Figures and Tables

**Table 1:** Comparison of the performance of nonenzymatic glucose sensors based on graphene and transition metals. CS: Chitosan; NPs: nanoparticles; GCE: glass carbon electrode; ILE: Ionic-liquid electrode; GR: grapheme.

Sensor	Detect Limit (μM)	Linear Range (mM)	Sensitivity (μA/mM∙cm^−2^)	Reference
AuNPs-graphene/ILE	0.062/0.183	/	97.8/16.3	[37]
GR-Nafion-PdNPs/GCE	1	0.01–5.0	/	[11]
CS-rGO–NiNPs/SPE	4.1	0.2–9.0	318.4	[24]
rGO-Ni(OH)_2_/GCE	0.6	0.002–3.1	11.43	[15]
NiO-GR/GCE	5	0.02–4.5	/	[38]
Ni/NiO-rGO-Nafion/SPE	1.8	0.03–6.44	1997	This work
